# Long non‐coding RNA NNT‐AS1 sponges miR‐424/E2F1 to promote the tumorigenesis and cell cycle progression of gastric cancer

**DOI:** 10.1111/jcmm.13726

**Published:** 2018-07-14

**Authors:** Beibei Chen, Qingfang Zhao, Lulu Guan, Huifang Lv, Liangyu Bie, Jinxi Huang, Xiao‐Bing Chen

**Affiliations:** ^1^ Department of Gastrointestinal Medical Oncology The Affiliated Cancer Hospital of Zhengzhou University Cancer Hospital of Henan Province Henan China; ^2^ Department of Gastrointestinal Surgery The Affiliated Cancer Hospital of Zhengzhou University Cancer Hospital of Henan Province Henan China

**Keywords:** cell cycle progression, E2F1, gastric cancer, miR‐424, NNT‐AS1

## Abstract

Long non‐coding RNAs (lncRNAs) have been illustrated to function as important regulators in carcinogenesis and cancer progression. However, the roles of lncRNA NNT‐AS1 in gastric cancer remain unclear. In the present study, we investigate the biological role of NNT‐AS1 in gastric cancer tumorigenesis. Results revealed that NNT‐AS1 expression level was significantly up‐regulated in GC tissue and cell lines compared with adjacent normal tissue and normal cell lines. The ectopic overexpression of NNT‐AS1 indicated the poor prognosis of GC patients. In vitro experiments validated that NNT‐AS1 knockdown suppressed the proliferation and invasion ability and induced the GC cell cycle progression arrest at G0/G1 phase. In vivo xenograft assay, NNT‐AS1 silencing decreased the tumour growth of GC cells. Bioinformatics online program predicted that miR‐424 targeted the 3′‐UTR of NNT‐AS1. Luciferase reporter assay, RNA‐immunoprecipitation (RIP) and RNA pull‐down assay validated the molecular binding within NNT‐AS1 and miR‐424, therefore jointly forming the RNA‐induced silencing complex (RISC). Moreover, E2F1 was verified to act as the target gene of NNT‐AS1/miR‐424, indicating the NNT‐AS1/miR‐424/E2F1 axis. In conclusion, our study indicates that NNT‐AS1 sponges miR‐424/E2F1 to facilitate GC tumorigenesis and cycle progress, revealing the oncogenic role of NNT‐AS1 for GC.

## 1 INTRODUCTION

Gastric cancer (GC) is one of the most common gastrointestinal cancers and ranks as the fifth most common cancers.^1^, ^2^, ^3^ Along with the progress of more comprehensive therapy techniques, the mortality rate of GC patients is decreasing in recent years; however, there are still huge quantity GC patients who are diagnosed at an advanced stage with lymphatic or distant metastasis in the absence of specific symptoms.^4^, ^5^ Because of the high rates of postsurgical recurrence and metastasis, the prognosis of GC patients diagnosed as advanced‐stage is pessimistically bad.^6^ Therefore, more attention should be devoted to the pathologic mechanism research.

Long non‐coding RNAs (lncRNAs) are a type of non‐coding RNAs (ncRNAs) with more than 200 nucleotides.^7^, ^8^ Non‐coding RNAs (ncRNAs) account for a large proportion of transcriptome, which are divided into short non‐coding RNAs and lncRNAs according to their length. Increasing evidences have indicated the extensive biological role of lncRNAs on multiple tumour pathological process, for example proliferation, invasion, metastasis and recrudesce.^9^, ^10^ For the tumorigenesis of GC, more and more lncRNAs have been identified to modulate the pathological process. For instance, long intergenic non‐coding RNA 01296 (LINC01296) acts as oncogenic lncRNA in GC carcinogenesis and aggravates gastric cancer cells progress via LINC01296/miR‐122/MMP‐9 regulatory pathway.^11^

In the pre‐experiments of our study, we focus on several candidate lncRNAs, and then we measured the expression levels of these candidate lncRNAs in cancer tissue using RT‐PCR. After comparing, we found that the lncRNA NNT‐AS1 expression level is up‐regulated in GC tissue samples and cells. NNT‐AS1 has been found to act as oncogene in human cancer.^12^ Besides, the roles and molecular mechanism of lncRNA NNT‐AS1 on GC tumorigenesis are unknown and never reported. Thus, we choose lncRNA NNT‐AS1 as our research object. In the present study, we aim to investigate the expression of lncRNA NNT‐AS1 in GC cells tumorigenesis. Besides, we analysed the relationship between NNT‐AS1 levels and the clinicopathological features of GC and investigated the biological effect and mechanisms of NNT‐AS1 on the phenotypes of GC cells in vitro and in vivo. Results indicate that NNT‐AS1 is a vital regulator in the cell cycle progression of GC through sponging miR‐424/E2F1.

## 2 MATERIALS AND METHODS

### 2.1 Patient samples

A total of 48 GC samples were collected from August 2015 to July 2017 at The Affiliated Cancer Hospital of Zhengzhou University. There are no any patients who had received preoperative chemotherapy before this study. The tissues were excised during the surgery and snap‐frozen in liquid nitrogen. This study was approved by the Ethic Committee of The Affiliated Cancer Hospital of Zhengzhou University. All written informed consents had been collected from every patient before surgery.

### 2.2 Cell culture and transfection

The human GC cell lines (SGC‐7901, BGC‐823, MGC‐803 and MKN‐45) were obtained from the Chinese Institute of Biochemistry and Cell Biology (Shanghai, China). Normal gastric mucosa cell line GES‐1 was purchased from American Type Culture Collection (ATCC, Rockville, MD, USA). Cell lines were cultured in Dulbecco's Modified Eagle's medium (DMEM, Invitrogen, Carlsbad, CA, USA) containing 10% fetal bovine serum (FBS, Gibco, USA) at 37°C in a humidified incubator containing 5% CO_2_. For transfection, miR‐424 mimics, miR‐424 inhibitor and siRNAs targeting NNT‐AS1 were provided by GenePharma (Shanghai, China) for silencing or up‐regulation. Transfection was performed using Lipofectamine2000 (Invitrogen, USA) following the instructions of manufacturer. All assays were repeated at least 3 times.

### 2.3 Quantitative reverse transcription polymerase chain reaction (RT‐PCR)

Total RNAs were extracted from the tumour tissue and adjacent normal tissues or cells using TRIzol reagent (Life Technologies, Carlsbad, CA, USA) following the manufacturer's protocol. PrimeScript RT Reagent Kit (TaKaRa, Dalian, China) was used to synthesize complementary DNA (cDNA). The qRT‐PCR was performed using SYBR Premix Ex Taq (TaKaRa). The primers for PCR were as follows: NNT‐AS1, forward 5′‐AGTTCCACCAAGTTTCTTCA‐3′, reverse 5′‐AGGTTTTGCCAGCATAGAC‐3′; miR‐424, forward, 5′‐GCTCGAGATATGGAGGGCGCC‐3′, reverse, 5′‐GGAACGGCAGACACGTA TCC‐3′. Each expression level was calculated with the 2^−▵▵Ct^ method and β‐actin acted as the endogenous control to normalize the data. The PCR was performed in triplicate.

### 2.4 Cell Counting Kit‐8 assay

Cell proliferation assay was performed with Cell Counting Kit‐8 (CCK‐8; Dojindo Laboratories, Kumamoto, Japan). GC cells (MGC‐803, SGC‐7901) were seeded into 96‐well plates at a density of 1 × 10^3^ per well. About 10 μL of CCK‐8 solution was added into each well at indicated time points. After incubation at 37°C for 2 hours, the absorbance was measured using an automatic microplate at 450 nm.

### 2.5 Transwell invasion assay

GC cells (MGC‐803, SGC‐7901) were transfected with indicated oligonucleotides. Then, 24‐well transwell chamber (Costar, MA, USA) with or without matrigel coating was performed for invasion. Briefly, GC cells (1 × 10^5^ cells per well) were suspended in serum‐free medium and transferred to the upper compartment which was added with 10% FBS while the lower compartment was filled with 600‐μL DMEM supplemented with 10% FBS. After incubation for 24 hours at 37°C, cells were fixed with ice‐cold methanol for 30 minutes and stained with 0.1% crystal violet for 10 minutes. The invaded cell number was determined in 5 randomly selected high‐power fields. Assay was performed in triplicate.

### 2.6 Western blot analysis

The expression of E2F1 protein in GC cells was detected by performing immunoblotting. Cells were lysed in RIPA buffer (pH 7.4) with protease and phosphatase inhibitors (Roche, Complete Mini). Equal amount of protein was loaded onto a SDS‐PAGE gel and transferred to PVDF membrane. The membrane was probed with the first antibody: E2F1 (1:1000, Abcam, MA, USA), CDK6 (1:1000, Abcam), Cyclin E (1:1000, Abcam), Cyclin D1 (1:1000, Abcam) and GAPDH (1:1000, Abcam) at 4°C overnight. Then, blots were incubated with HRP‐conjugated secondary antibody (1:5000). The antibodies were detected using enhanced chemiluminescence reagent (32109; Thermo Fisher Scientific).

### 2.7 Luciferase reporter assay

For Luciferase assays, NNT‐AS1 wild type with potential miR‐424 binding sites or mutant sites was generated by Sangon Biotech (Shanghai, China), and then fused with the luciferase reporter vector psi‐CHECK‐2 (Promega, Madison, WI, USA). SGC‐7901 cells (2 × 10^4^ cells/well) were seeded into 96‐well plates. Cells were co‐transfected with luciferase plasmids and miRNA mimics or controls. Cells were cultured in 12‐well plates and transfected with 5 ng plasmid and 5 ng Renilla using Dual‐Luciferase Reporter Assay System (Promega). Every analysis was performed 3 times.

### 2.8 RNA immunoprecipitation (RIP)

RNA immunoprecipitation (RIP) assays were performed as previously described.^13^ Wild‐type NNT‐AS1 or mutant NNT‐AS1 were synthesized and then cloned into SGC‐7901 cells. SGC‐7901 cells were also co‐transfected with miR‐NC or miR‐424 mimic. Cells were lysed by lysis buffer containing a protease inhibitor cocktail and RNase inhibitor. Magnetic beads were pre‐incubated with an anti‐GFP antibody (Abcam) or anti‐rabbit IgG (Millipore) for 1 hour at room temperature, and lysates were immunoprecipitated with beads at 4°C overnight. After 48 hours, cells were used to perform RIP assay using an anti‐AGO2 antibody (Millipore) as described above.

### 2.9 RNA pull‐down assay

RNA pull‐down assay was performed using Magnetic RNA‐Protein Pull‐Down Kit (Pierce, USA) according to the manufacturer's instructions. Briefly, miRNA was labelled using biotin and then transfected into SGC‐7901 cells. A biotinylated miRNA without complementary sites with NNT‐AS1 acted as negative control. After 48 hours, cells were harvested for biotin‐based pull‐down assay. The expression or enrichment was measured using RT‐PCR.

### 2.10 Xenograft in vivo mice assay

Ten athymic BALB/C nude mice (4‐week old, 8‐10 g) were all purchased from Shanghai Experimental Animal Center of Chinese Academy of Sciences (Shanghai, China). SGC‐7901 cells (3 × 10^6^) transfected with lentivirus‐mediated NNT‐AS1 shRNA (sh‐NNT‐AS1) or negative control (sh‐NC) were subcutaneously injected into nude mice. Tumour volumes were measured every 3 days and calculated according to 0.5 × length × width^2^. The in vivo assay was performed following the approval by the Animal Care and Experiment Committee of The Affiliated Cancer Hospital of Zhengzhou University.

### 2.11 Statistical analysis

Statistical analyses and data calculation were performed using the SPSS19.0 (IBM, SPSS, Chicago, IL, USA) software and graphed using GraphPad Prism 6 (GraphPad Software, San Diego, CA, USA). Overall survival curves were calculated using Kaplan‐Meier analysis and the log‐rank test. Pearson correlation coefficient was used to analyse the correlations. Comparisons between groups were analysed by the *t* tests χ^2^ test, one‐way analysis of variance (ANOVA). The differences were considered statistically significant at *P* < .05.

## 3 RESULTS

### 3.1 LncRNA NNT‐AS1 was highly expressed in GC tissues and cells, and indicated poor prognosis of GC patients

**Figure 1:**
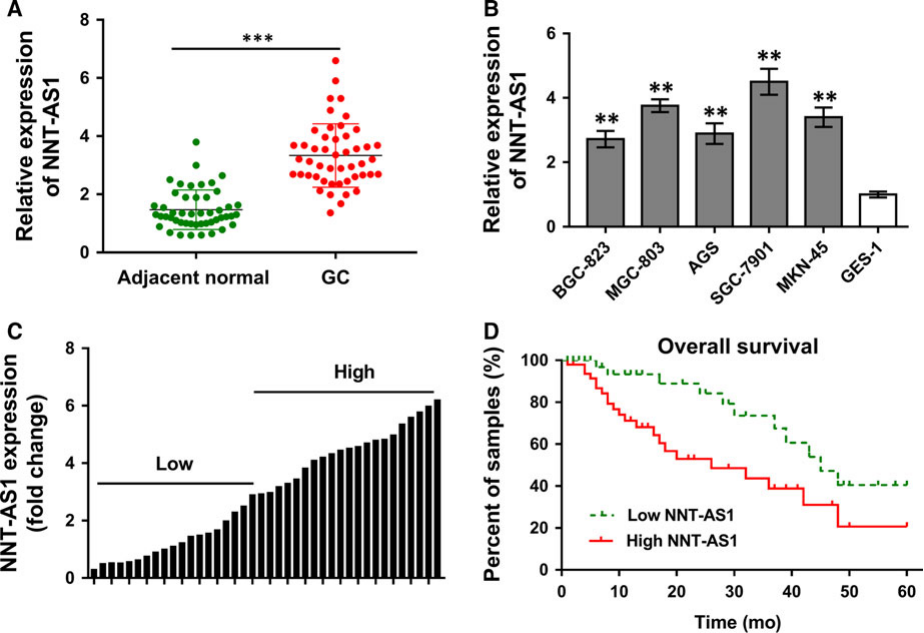
LncRNA NNT‐AS1 was highly expressed in GC tissues and cells, and indicates poor prognosis of GC patients. A, RT‐PCR revealed the expression levels of NNT‐AS1 in 48 GC samples collected in the research cohort. B, RT‐PCR revealed the NNT‐AS1 expression levels in GC cell lines (BGC‐823, MGC‐803, AGS, SGC‐7901, MKN‐45) compared with normal gastric mucosa cell line (GES‐1). C, According to median value of NNT‐AS1 expression, the whole group was divided into high NNT‐AS1 expression group (n = 21) and low NNT‐AS1 expression group (n = 27). D, Kaplan‐Meier curves and log‐rank test revealed the overall survival rate of GC patients with high/low NNT‐AS1 levels. *** *P* < .001, ** *P* < .01 compared to control group

To verify the expression of NNT‐AS1 in GC tumour tissue and cells, RT‐PCR was performed on these collected samples and cultured cells. The clinicopathologic data of GC patients were presented in Table 1. Results revealed that NNT‐AS1 expression was significantly up‐regulated in these collected GC tissue samples compared with adjacent normal tissue (Figure 1A). Meanwhile, RT‐PCR revealed that NNT‐AS1 expression was also significantly up‐regulated in GC cell lines (BGC‐823, MGC‐803, AGS, SGC‐7901, MKN‐45) compared with normal gastric mucosa cell line (GES‐1) (Figure 1B). According to median value of NNT‐AS1 expression, the whole group was divided into high NNT‐AS1 expression group (n = 21) and low NNT‐AS1 expression group (n = 27) (Figure 1C). Overall survival rate of GC patients was calculated using Kaplan‐Meier curves and log‐rank test, showing that GC patients with high NNT‐AS1 levels had poor prognosis than those with low NNT‐AS1 level (Figure 1D). In summary, the data concluded that lncRNA NNT‐AS1 was highly expressed in GC tissues and cells, and the ectopic overexpression indicated poor prognosis of GC patients.

**Table 1:** Relationship between NNT‐AS1 expression and clinicopathological characteristics of GC patients

	Case (48)	NNT‐AS1		*P* value
		Low (N = 21)	High (N = 27)	
Age				
<60	26	11	15	.705
≥60	22	10	12	
Gender				
Male	28	12	16	.694
Female	20	9	11	
Histological grade				
Well/moderate	16	7	9	.114
Poor/other	32	14	18	
T stage				
T1‐T2	29	14	15	.022^a^
T3‐T4	19	7	12	
Lymphatic metastasis				
Present	35	18	17	.002^a^
Absent	13	3	10	
TNM stage				
I‐II	18	8	10	.003^a^
III‐IV	30	13	17	

TNM, tumor‐node‐metastasis; Well, Well‐differentiated adenocarcinoma; Moderate, moderately differentiated adenocarcinoma; Poor, poorly differentiated adenocarcinoma; Other, other histological type.

a *P* < .05 represents statistical differences.

### 3.2 NNT‐AS1 knockdown induced the GC cell cycle progression arrest at G0/G1 phase

**Figure 2:**
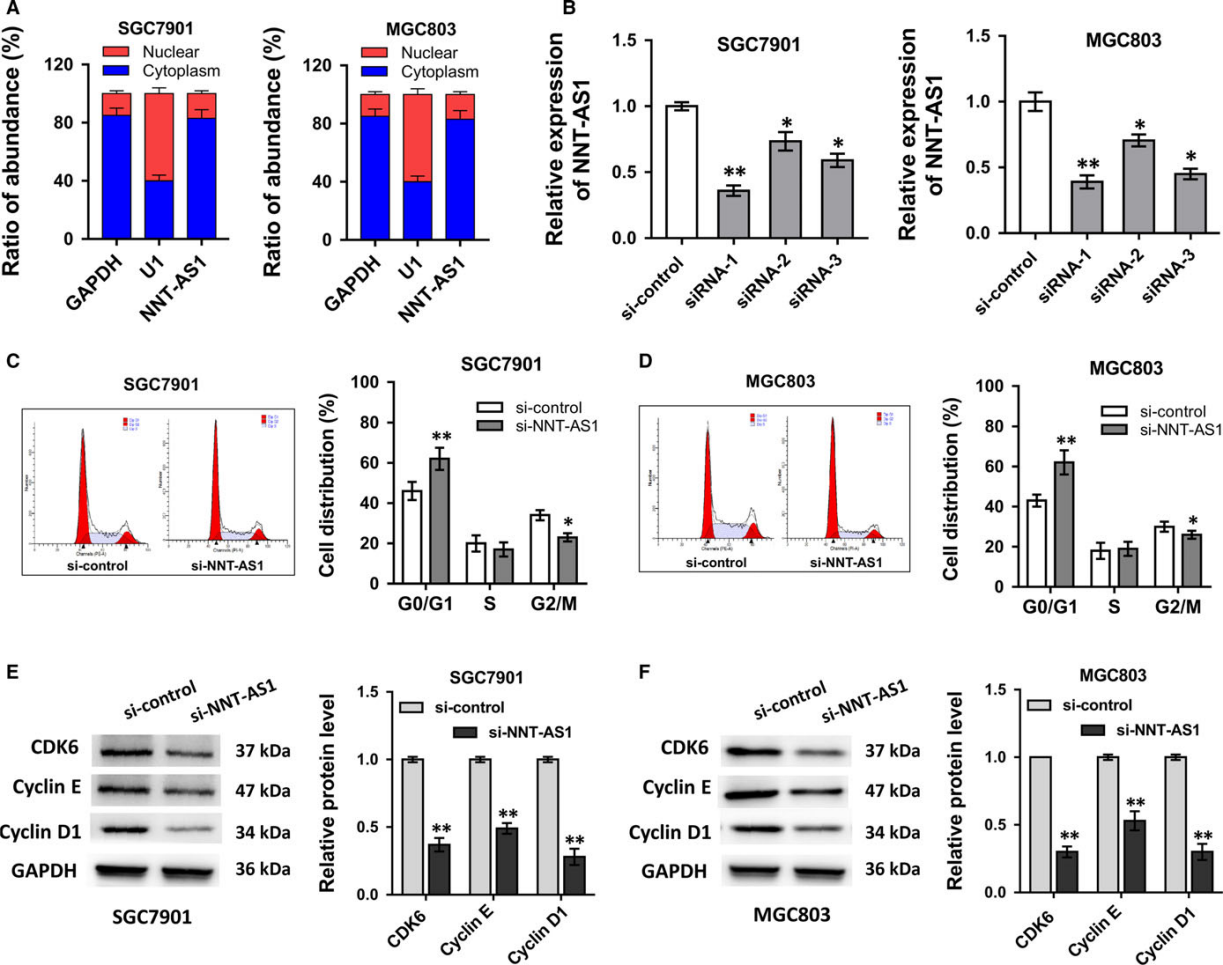
NNT‐AS1 knockdown induced the cell cycle progression arrest at G0/S phase. A, The expression levels of NNT‐AS1 in the nucleus and cytoplasm of GC cells (SGC‐7901, MGC803). U1 (nuclear retained) and GAPDH (exported to cytoplasm) were used as controls. B, Small interfering RNAs (siRNAs) targeting NNT‐AS1 were transfected into GC cell lines (SGC‐7901, MGC803). NNT‐AS1 expression was measured by RT‐PCR. C, D, RT‐PCR showed the cell cycle distribution of GC cell lines (SGC‐7901, MGC803) transfected with si‐NNT‐AS1 and si‐control. E, F, Western blot analysis revealed the cycle‐related protein levels of CDK6, Cyclin E and Cyclin D1, in GC cell lines (SGC‐7901, MGC803). ** *P* < .01, *P < .05 compared to control group

Although the ectopic overexpression of lncRNA NNT‐AS1 had been identified in GC tissues and cells, the biologic role of it on GC tumorigenesis was still ambiguous. RT‐PCR revealed that the cellular localization of NNT‐AS1 was mainly in cytoplasm, instead of nuclear (Figure 2A). Small interfering RNAs (siRNAs) targeting NNT‐AS1 were transfected into GC cell lines (SGC‐7901, MGC803) to decrease the expression levels of NNT‐AS1 (Figure 2B). Cell cycle distribution measured by flow cytometry analysis showed that NNT‐AS1 knockdown increased the cell distribution at G0/G1 phase, suggesting the cycle progression arrest at G0/G1 phase (Figure 2C, D). Western blot analysis revealed that NNT‐AS1 knockdown significantly decreased the cycle‐related protein levels of CDK6, Cyclin E and Cyclin D1, compared with control transfection in GC cell lines (SGC‐7901, MGC803) (Figure 2E, F). Overall, the above data concluded that NNT‐AS1 was mainly located in the cytoplasm, and NNT‐AS1 knockdown induced the GC cell cycle progression arrest at G0/G1 phase.

### 3.3 NNT‐AS1 knockdown suppressed the proliferation and invasion ability in vitro, and inhibited the GC tumour growth in vivo

**Figure 3:**
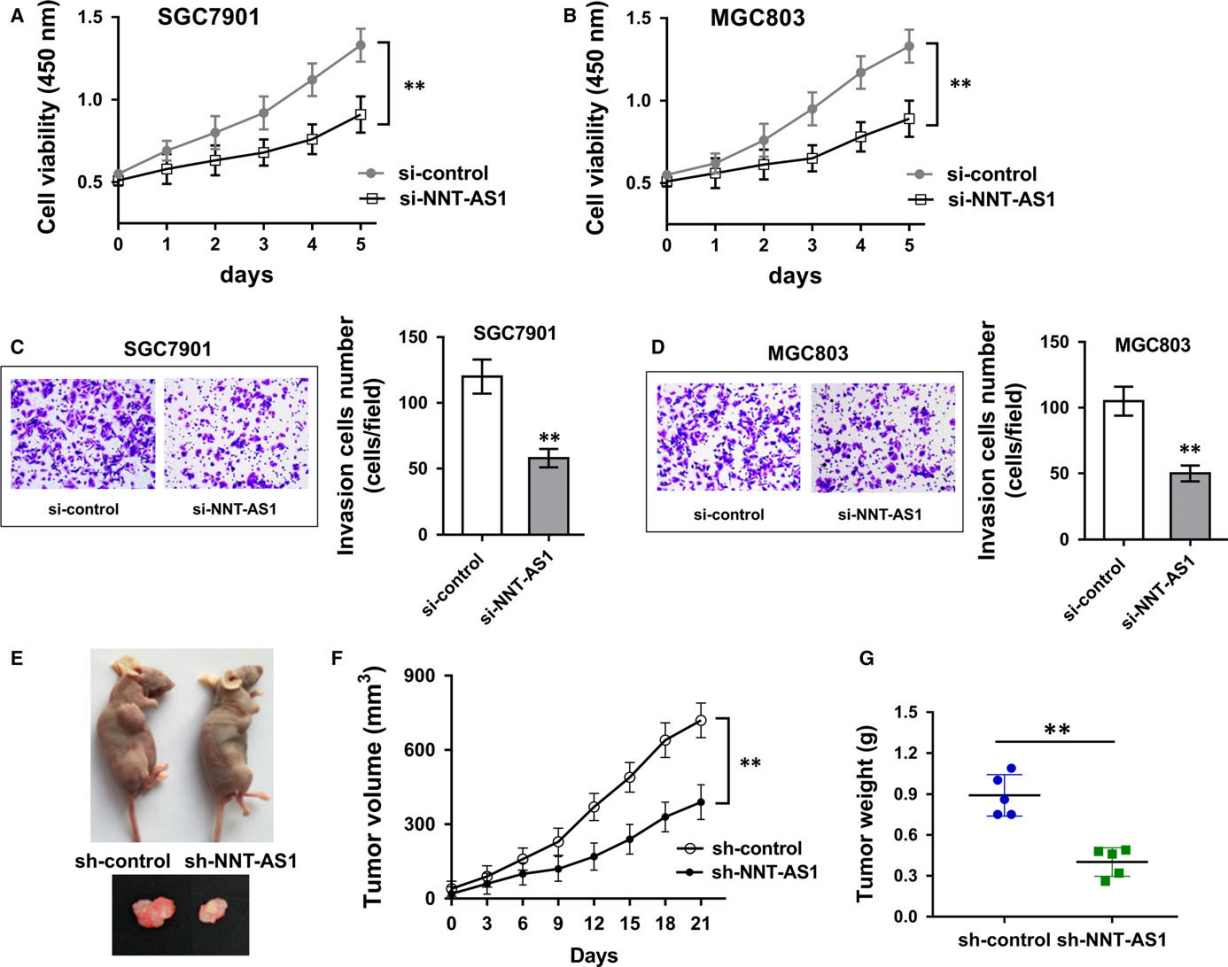
NNT‐AS1 knockdown suppressed the proliferation and invasion ability in vitro, and inhibited tumour growth in vivo. A, B, CCK‐8 assay showed the proliferation ability of GC cell (SGC‐7901, MGC803) transfected with si‐NNT‐AS1 or si‐control. C, D, Transwell invasion assay showed the invaded cell number in GC cell (SGC‐7901, MGC803) transfected with si‐NNT‐AS1 or si‐control. E, Images of xenograft in vivo assay. F, G, The tumour volume and weight of GC cells in mice injected with SGC‐7901 cells transfected with lentivirus‐mediated transfection shRNA or controls. **P < .01 compared to control group

Subsequently, we tested the role of NNT‐AS1 knockdown on GC tumour phenotype using in vitro and in vivo experiments. CCK‐8 assay showed that NNT‐AS1 knockdown suppressed the proliferation ability of GC cell (SGC‐7901, MGC803) compared with control group (Figure 3A, B). Transwell invasion assay showed that NNT‐AS1 knockdown inhibited the invasive cells compared with control groups (Figure 3C, D). Xenograft in vivo assay was performed to verify the function of NNT‐AS1 on GC tumour growth (Figure 3E). Results showed that NNT‐AS1 knockdown induced by lentivirus‐mediated transfection significantly decreased the tumour volume of GC cells (Figure 3F), besides suppressing the tumour weight after killing (Figure 3G). In summary, the above results concluded that NNT‐AS1 knockdown suppressed the proliferation and invasion ability of GC cells in vitro, and inhibited the GC tumour growth in vivo.

### 3.4 NNT‐AS1 targeted miR‐424 as miRNA ‘sponge’

**Figure 4:**
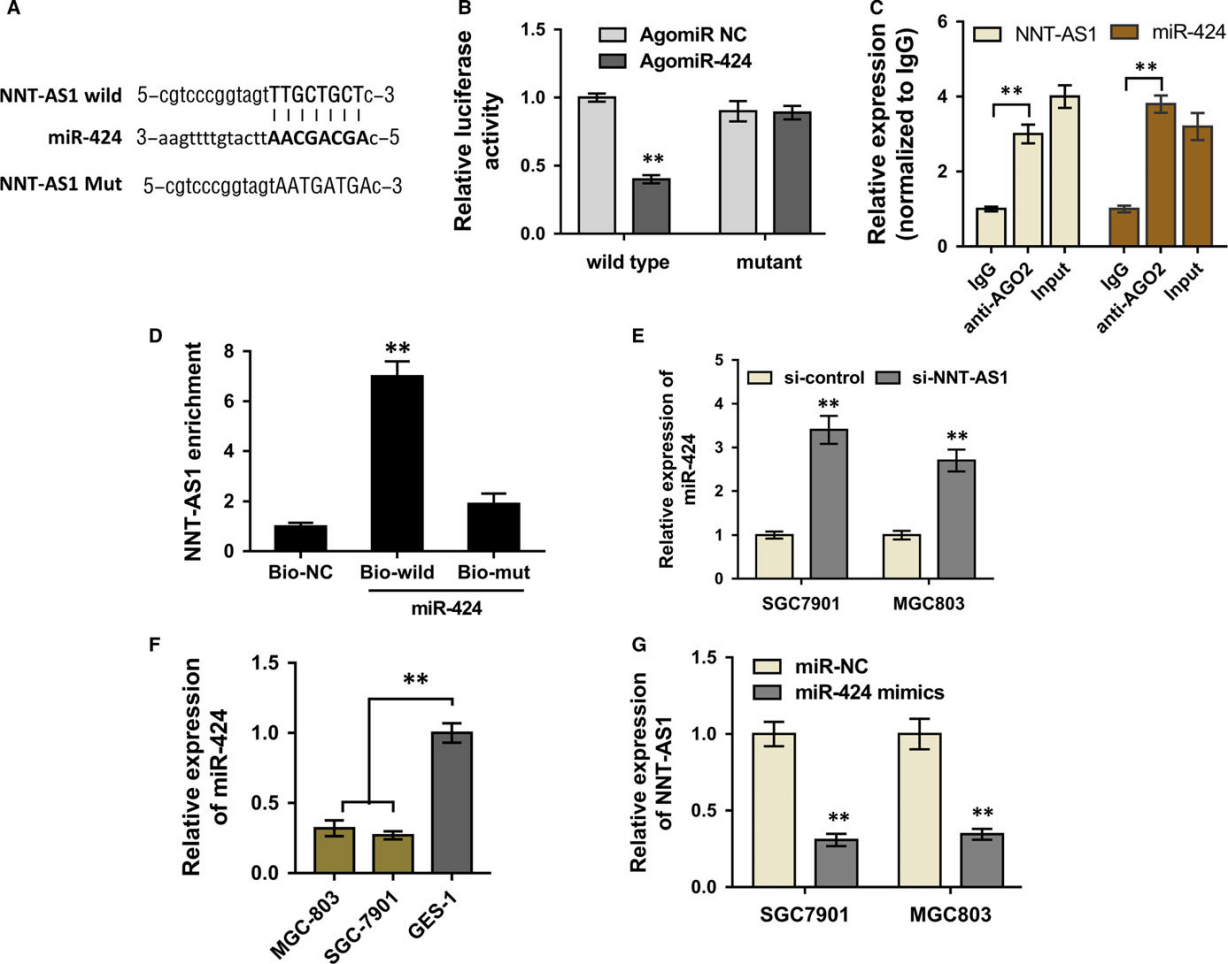
NNT‐AS1 targeted miR‐424 at 3′‐UTR with complementary binding sites. A, The predicted complementary binding sequences of NNT‐AS1 3′‐UTR and miR‐424. B, Luciferase reporter assay showed the molecular binding within NNT‐AS1 and miR‐424. C, RIP assay was carried out to explore the exact mechanism of the interaction between NNT‐AS1 and miR‐424, indicating that both NNT‐AS1 and miR‐424 were significantly enriched in Ago2 immunoprecipitate compared to IgG‐pellet. D, miR‐424 directly binds with NNT‐AS1 using pull‐down assay. SGC‐7901 cells were transfected with biotinylated wild‐type/mutant‐type miR‐424, and after 48 h, NNT‐AS1 expression levels were measured using RT‐PCR. E, RT‐PCR showed that miR‐424 expression was increased in GC cell lines (MGC‐803, SGC‐7901) transfected with si‐NNT‐AS1 or control. F, RT‐PCR showed the expression of miR‐424 in GC cell lines (MGC‐803, SGC‐7901) compared to normal gastric mucosa cell line (GES‐1). G, RT‐PCR showed the expression of NNT‐AS1 in GC cell lines (MGC‐803, SGC‐7901) transfected with miR‐424 mimics or control. **P < .01 compared to control group

Prior assay revealed that the cellular localization of NNT‐AS1 was mainly in the cytoplasm, instead of nuclear (Figure 2A). Therefore, we assumed that NNT‐AS1 might function as miRNA ‘sponge’ in the GC tumorigenesis. Bioinformatics online program (StarBase, http://starbase.sysu.edu.cn) predicted that miR‐424 shared with complementary binding sites with NNT‐AS1 at 3′‐Untranslated Regions (3′‐UTR) (Figure 4A). Luciferase reporter assay showed that NNT‐AS1 combined with miR‐424 at 3′‐UTR with molecular binding (Figure 4B). RIP assay indicated that both NNT‐AS1 and miR‐424 were significantly enriched in Ago2 immunoprecipitate compared to IgG‐pellet, suggesting that both NNT‐AS1 and miR‐424 were in the same RNA‐induced silencing complex (RISC) (Figure. 4C). Besides, biotin‐avidin pull‐down assay was performed to determine whether miR‐424 could directly bind with NNT‐AS1. Results showed that miR‐424 directly binds with NNT‐AS1 (Figure 4D). RT‐PCR showed that miR‐424 was significantly overexpressed in GC cell (SGC‐7901, MGC803) transfected with si‐NNT‐AS1 or si‐NC (Figure 4E). Meanwhile, miR‐424 expression was significantly down‐regulated in GC cell lines (MGC‐803, SGC‐7901) when compared with normal gastric mucosa cell line (GES‐1) (Figure 4F). Moreover, NNT‐AS1 expression was also significantly down‐regulated in GC cell lines (MGC‐803, SGC‐7901) transfected with miR‐424 mimics or control (Figure 4G). Overall, the results concluded that NNT‐AS1 targeted miR‐424 with complementary binding sites, acting as a miRNA ‘sponge’.

### 3.5 NNT‐AS1/miR‐424 targeted E2F1 in the cycle progression regulation of GC cells

**Figure 5:**
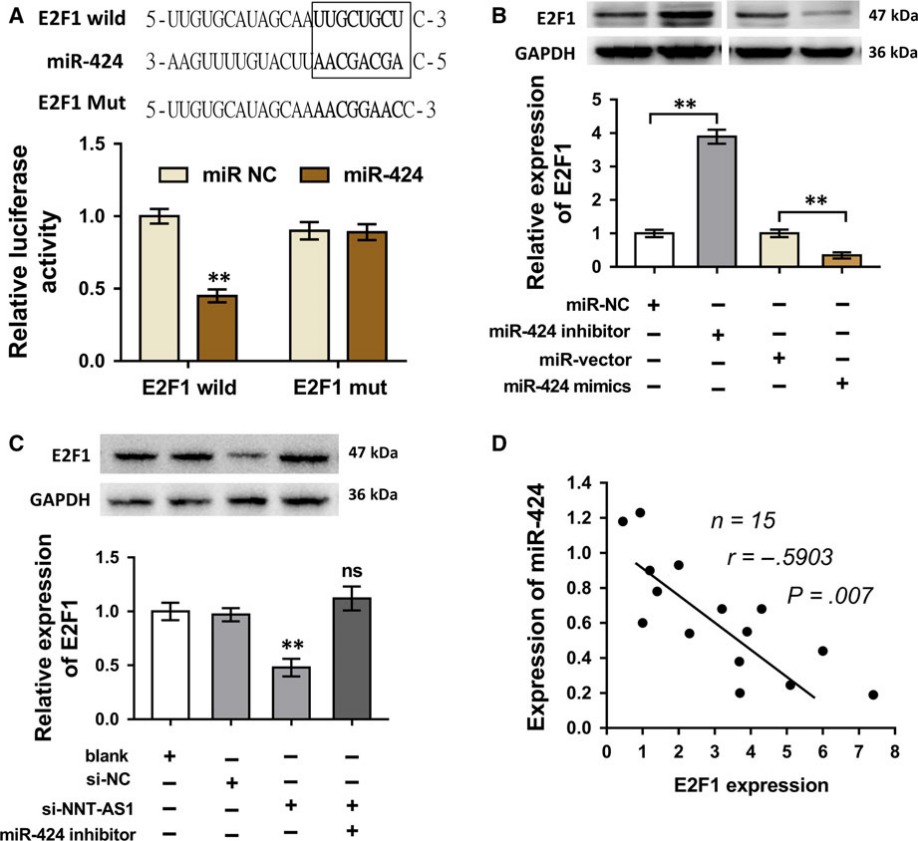
NNT‐AS1/miR‐424 targeted E2F1 in the cycle progression regulation of GC cells. A, Bioinformatics programs (TargetScan, http://www.targetscan.org) predicted the complementary binding sites within miR‐424 and E2F13′‐UTR. The prediction was confirmed by luciferase report assay. B, Western blot assay showed the E2F1 protein expression in SGC‐7901 cells transfected with miR‐424 inhibitor or miR‐424 mimics. C, Western blot assay showed the E2F1 protein expression in SGC‐7901 cells transfected with si‐NC, si‐NNT‐AS1 or/and miR‐424 inhibitor. D, Pearson's correlation showed negative correlation with E2F1 mRNA and miR‐424 in 15 cases of GC patient samples. **P < .01 compared to the corresponding control group

Moreover, bioinformatics programs (TargetScan, http://www.targetscan.org) predicted that miR‐424 targeted the 3′‐UTR of E2F1 with complementary binding sites (Figure 5A). Moreover, luciferase reporter assay confirmed the molecular interaction within miR‐424 and E2F1 mRNA. In SGC‐7901 cells, Western blot assay showed that E2F1 protein expression was significantly up‐regulated when transfected with miR‐424 inhibitor, while it was down‐regulated when transfected with miR‐424 mimics (Figure 5B). Furthermore, in SGC‐7901 cells, Western blot assay validated that E2F1 protein expression was decreased when transfected with si‐NNT‐AS1, which was reversed by the co‐transfection of miR‐424 inhibitor (Figure 5C). Pearson's correlation analysis revealed that E2F1 expression was negatively correlated to miR‐424 expression in 15 cases of GC patients samples (Figure 5D). Overall, these data concluded that NNT‐AS1/miR‐424 targeted E2F1 protein expression in the cycle progression regulation of GC cells in vitro.

## 4 DISCUSSION

Long non‐coding RNAs have recently emerged as an important regulator on transcriptional and post‐transcriptional regulation of several diseases, including cancers.^14^, ^15^, ^16^ LncRNA NNT‐AS1 has been found to participate in the tumorigenesis of hepatocellular carcinoma, cervical cancer and colorectal cancer.^17^, ^18^, ^19^ In the pre‐experiments of our study, we found that the lncRNA NNT‐AS1 expression level was up‐regulated in GC tissue samples and cells. Besides, the roles and molecular mechanism of lncRNA NNT‐AS1 on GC tumorigenesis are unknown and never reported. Thus, we choose lncRNA NNT‐AS1 as our research object. In the present study, our study investigates the role of lncRNA NNT‐AS1 in gastric cancer (GC) tumorigenesis and identifies the underlying mechanism.

During the carcinogenesis of GC, a great deal of lncRNAs might be abnormally expressed.^20^, ^21^ Generally, the significantly dysregulated lncRNAs might involve the pathological process and exert vital roles by regulating multiple progression. In our study, we found that lncRNA NNT‐AS1 was significantly high‐expressed in gastric cancer tissue and cell lines when compared with adjacent normal tissue and normal cells. Besides, the ectopic overexpression of NNT‐AS1 indicates poor prognosis of GC patients calculated using Kaplan‐Meier curves and log‐rank test. Therefore, the evidence proved the oncogenic role of NNT‐AS1 in GC tumorigenesis.

The oncogenesis of GC is a complex pathophysiological process involved in multifarious pathophysiological process, including proliferation, invasion, metastasis and cycle progression.^22^, ^23^ In subsequent functional experiments in vitro, results revealed that NNT‐AS1 knockdown suppressed the proliferation and invasion ability in vitro, induced the GC cell cycle progression arrest at G0/G1 phase and inhibited the GC tumour growth in vivo. Bioinformatics online tools predicted the complementary binding within NNT‐AS1 and miR‐424. Luciferase reporter assay confirmed the molecular binding at 3′‐UTR. Moreover, RIP assay indicated that NNT‐AS1 and miR‐424 form the RNA‐induced silencing complex (RISC). Therefore, we validate the direct molecular interaction within NNT‐AS1 and miR‐424, illustrates that NNT‐AS1 functions as miR‐424 ‘sponge ‘.

Long non‐coding RNAs are a type of non‐coding RNA (ncRNA) without protein‐coding ability.^24^, ^25^ Up to now, the most common regulatory mechanism for lncRNA is the miRNA ‘sponge’ theory. In our study, our data indicate that NNT‐AS1 harbours miR‐424 by 3′‐UTR binding, while miR‐424 also targets the 3′‐UTR of E2F1 mRNA. E2F1 protein is an important transcription factor among E2F family, which is a vital regulator in cell cycle regulation and apoptosis. Emerging evidences have proved that E2F1 closely controls the cellular process from G1 to S phase, acting as a critical element for cycle progression. In addition, previous results reveal that NNT‐AS1 knockdown suppressed the cycle‐related protein levels of CDK6, Cyclin E and Cyclin D1 in GC cell lines. Besides, NNT‐AS1 knockdown induced G0/G1 phase arrest. Based on the above findings, we conclude that NNT‐AS1 exerts the cycle inhibitive function through targeting miR‐424/E2F1 axis. E2F1 is found to be aberrantly high‐expressed in gastric cancer, and enhanced E2F1 expression promotes proliferation, while E2F1 low expression decreased cell proliferation by blocking cell cycle in GC cells.^26^ Moreover, R‐424‐5p is found to be up‐regulated in GC tissues and cells, and its high‐expression could promote the proliferation of GC cells.^27^

More than NNT‐AS1, other lncRNAs also modulate the cellular process of GC cells.^28^ For example, lncRNA SNHG6 is overexpressed in gastric cancer tissues and cell lines, and SNHG6 could epigenetically silence p27 and competitively sponge miR‐101‐3p to regulate ZEB1.^29^ In HCC tumorigenesis, NNT‐AS1 knockdown inhibited the HCC cells progression via miR‐363 thereby targeting CDK6 expression.^18^ Therefore, our study firstly confirmed the oncogenic role of lincRNA NNT‐AS1 in GC tumorigenesis, revealing the vital pathway of NNT‐AS1/miR‐424/E2F1 axis.

In summary, our study validated that the overexpression of NNT‐AS1 is observed in GC tissue and cells. The ectopic high‐expressed NNT‐AS1 indicated the poor prognosis of GC patients. On the molecular level, NNT‐AS1 promotes the oncogenesis and cycle progress of GC cells via miR‐424/E2F1 axis.

## CONFLICTS OF INTEREST

All the authors declare that they have no competing interests.
